# Four-color fluorescence in-situ hybridization is useful to assist to distinguish early stage acral and cutaneous melanomas from dysplastic junctional or compound nevus 

**DOI:** 10.1186/s13000-020-00937-9

**Published:** 2020-05-11

**Authors:** Yumei Lai, Yan Wu, Ruping Liu, Aiping Lu, Lixin Zhou, Lin Jia, Xinting Diao, Zhongwu Li

**Affiliations:** 1 Department of Pathology, Peking University Cancer Hospital & Institute, Key Laboratory of Carcinogenesis and Translational Research (Ministry of Education/Beijing), Beijing, 100142 People’s Republic of China; 2 Beijing Institute of Graphic Communication, Beijing, 102600 People’s Republic of China

**Keywords:** Acral, Dysplastic nevus, Early stage, Fluorescence in-situ hybridization, Melanoma

## Abstract

**Background/objective:**

Acral and cutaneous melanomas are usually difficult to accurately diagnose in the early stage, owing to the similarity in clinical manifestations and morphology with those of dysplastic nevus (DN). In this study, we aimed to evaluate the diagnostic value of four-color fluorescence in-situ hybridization (FISH) probes specific to the *RREB1*,*CCND1*,and *MYB* genes, and centromere of chromosome 6, in distinguishing DN and melanoma.

**Methods:**

Fifty one DN and 58 melanoma cases were collected and tested with four-color FISH. Histological features were reviewed and concordant morphologic diagnosis by three pathologists was considered the golden criterion.

**Results:**

Fifty DN and 59 melanoma cases, with 37 melanomas in situ and 22 melanomas in Clark level 2, were confirmed finally; among them, 42 (71.2%) cases were acral. A comparison of clinicopathological features between the two entities showed that several features were considerably more frequently observed in the melanoma group, including more mitotic figures, stratum corneum pigmentation, lymphocyte infiltration, cell atypia, successive or pagetoid melanocyte growth pattern in the epidermis, larger tumor size, and older age at diagnosis. FISH was positive in 3 (6.0%) DN and 56 (94.9%) melanoma cases according to Gerami’s criteria. In distinguishing the two groups, the sensitivity of the four-color FISH was 94.9% and specificity was 94.0%.We found that *CCND1* gain was the most sensitive, either in Gerami’s or Gaiser’s criteria. Further analysis showed that *CCND1*gain was more obvious in the acral group of melanoma.

**Conclusions:**

We conclude that the four-color FISH test was highly sensitive and specific in distinguishing early-stage acral and cutaneous melanomas from dysplastic nevus in Chinese population, and the most sensitive criterion was the gain of *CCND1*.

## Background

It is usually challenging for pathologists to diagnose melanoma in the early stage, especially to discriminate melanoma in situ from dysplastic nevus (DN); moreover, it is hard to reach consensus among pathologists, even among the experts. This is because there is no single criterion that can absolutely distinguish melanoma from DN and there are several overlapping clinical and histopathological features of the two entities. In a retrospective study performed by the Dutch Melanoma Working Party in Holland on 1069 melanocytic lesions diagnosed by local doctors, 8% could not be accurately diagnosed and 14% cases with initial diagnosis of melanoma were actually benign nevi, while 17% of cases with initial diagnosis of benign nevi turned out to be malignant later [1]. With respect to some ambiguous melanocytic lesions, neither clinicians nor pathologists can make a clear judgment [2]_._ On the contrary, patients might sustain physical and mental injuries due to under or over treatment.

Diagnosing melanoma using four-color fluorescence in-situ hybridization (FISH) has been proven to be effective [3–11]. The four-color FISH probes for 6p25 (*RREB1*-Ras responsive element-binding protein-1), 6q23 (*MYB*-myeloblastosis), 11q13 (*CCND1*,cyclin-D1 or chromosome 11q), and *CEP6* (a centromeric reference point on chromosome 6) distinguished melanoma and benign melanocytic lesions according to variation in copy number of the genes, usually gain. Furthermore, the results were highly consistent with histological diagnosis, with the sensitivity of 70.5–100% and specificity of 90–100% [3–11]. However, data about its significance in the diagnosis of early-stage melanoma and DN are limited, probably because of limited cases of the two entities and difficulty in diagnosis. Furthermore, data on early lesions in acral has not been reported. In this study, we aimed to evaluate the diagnostic value of four-color fluorescence in-situ hybridization (FISH) probes in distinguishing DN and melanoma.

## Materials and methods

### Patients and inclusion criteria

One hundred and nine surgical excision specimens, 51 DN and 58 early-stage melanoma specimens, either acral or cutaneous, were collected from January 1, 2008 to December 31, 2017 in the Pathology Department of the Peking University Cancer Hospital in Beijing, China. All samples were fixed with neutral buffered formalin, embedded in paraffin, and reviewed by three experienced pathologists. Dysplastic nevus was defined based on the criteria of the Europe Organization for Research and Treatment of Cancer [12]. Specifically, a DN should fulfill at least three of the following four criteria: obvious hyperplasia of nevus cells in the basal layer, irregular nests of nevus cells, nevus cells with a large nucleolus, and nevus cells with lymphocytes or histocytes commonly seen in the background. Furthermore, for all the melanoma cases, Clark level was confined to no more than level 2, that is, only a small part of the papillary layer of dermis was invaded.

### Histopathological diagnosis and morphological parameters

The golden standard to evaluate the sensitivity and specificity of FISH was determined as the histopathological diagnosis by three experienced pathologists blinded to each other and to the results of FISH. For discordant diagnosis, an agreement of two pathologists was considered to be the final diagnosis. Meanwhile, morphologic features including melanocyte growth pattern, cell atypia, maturation, mitotic figures per square millimeter, stratum corneum pigmentation, and lymphocyte infiltration in the interstitial background of the papillary layer were observed under a light microscope, by hematoxylin and eosin (H&E) staining. Furthermore, lymphocyte infiltration was further interpreted as mild, moderate, and massive.

### FISH detection

For each case, the most suspicious area of 0.5 cm in diameter was selected according to H&E staining results for hybridization using the Vysis Melanoma FISH Probe Kit (Abbott Molecular Inc., Abbott Park, IL, USA) specific to *RREB1*,*CCND1*, *MYB*, and centromere of chromosome 6(*CEP6*). Specifically, FISH was performed with 4-μm-thick paraffin sections. The slides were baked at 60 °C overnight, and then deparaffinized with xylene for 10 min three times, dexylened with 100% ethanol for 5 min two times, and washed with water two times. Subsequently, the slides were pretreated with 10 mM citric acid buffer (Pretreatment Solution, Vysis, USA) at 80 °C for 12 min after washing with water for three times, followed by pepsin digestion at 37 °C for 25 min, water wash for two times, and dehydration in an ethanol series for 1 min each. Then, 10μLof probes were added to the tissues and denatured at 75 °C for 5 min and hybridized at 37 °C for 16 h. Thereafter, the slides were washed with 2_SSC/0.3% NP40 at 71 °C for 2 min, dehydrated naturally in dark, and counterstained with 10 μL of 4,6-diamino-2-phenylindole (DAPI, Vysis, USA). Finally, the slides were stored at − 20 °C after placing over glasses until observation.

FISH was analyzed by a trained physician, who signed the routine cytogenetic reports and was blinded to the results of histological diagnosis. The results were scored according to Gerami’s criteria [4]. That is, for each sample, 30 non-overlapping nuclei of tumor cells were counted and considered to be positive if one or more of the following four criteria was satisfied: more *RREB1* (6p25) copies than *CEP6* in more than 55% of cells (gain of *RREB1* relative to *CEP6*), over two copies of *RREB1* (6p25) in more than 29% of cells (gain of *RREB1*), less *MYB* (6q23) copies than *CEP6* in more than 40% of cells (loss of *MYB* relative to *CEP6*), and over two copies of *CCND1* (11q13) in more than 38% of cells (gain of *CCND1*).

Besides, the criteria of Gerami were compared with those of Gaiser [13], which included another four criteria, and the result was positive if any of the following criteria was fulfilled: an average of more than 2.5 copies of *CCND1*per cell, an average of more than 2.5 copies of *MYB* per cell, aberrant copies of *RREB1* in more than 63% of cells, and less *MYB* (6q23) copies than *CEP6* in more than 31% of cells.

### Statistical analysis

Difference in the age at onset between the two groups of melanoma and nevus and comparison of *CCND1* amplification in the mean *CCND1* copy number per cell and percentage of cell with more than two copies of *CCND1* between acral and cutaneous melanomas were compared using an independent *t*-test. Other differences between the melanoma and nevus groups, or between the acral and cutaneous groups, were analyzed using Pearson’s chi-square test. The results with *p* value of <.05 were considered statistically significant. Statistical analyses were performed using SPSS 19.0 for Windows (SPSS Inc., Chicago, IL, USA).

## Results

### Clinicopathological features evaluation

After the review by the three pathologists, two cases of melanoma in situ initially diagnosed were found to be dysplastic junctional nevi, while three cases of nevi were revised to be melanoma in situ. Finally, 50 cases of DN and 59 cases of melanoma were confirmed. There were 28 cases of compound nevi and 22 cases of junctional nevi. Among the 59 melanoma cases, 37 were melanoma in situ and 22 were level 2 according to Clark Staging, with Breslow thickness of 0.3–1.0 mm, and without any ulceration. Histological subtypes of the 22 invasive melanomas included 15 acral lentiginous melanomas, 7 superficial spreading melanomas, 1 lentigo maligna melanoma, and 1 spitzoid melanoma.

Clinicopathological features were compared between the melanoma and DN groups and the data are shown in Table 1.The gender ratio of the two entities showed no obvious difference, and so did the tumor site, with the most common sites being acral in both groups. Half of the DNs were more than 6.00 mm in diameter with the mean diameter of the group being 7.30 mm. However, compared with that in the DN group, the average tumor size in the melanoma group was significantly larger (mean diameter, 11.34 vs. 7.30 mm, *p* = .002), and the age at diagnosis was considerably older (mean age, 47.73 vs. 36.60 years, *p* < .001).

**Table 1:** Clinicopathological feature of dysplastic nevi and early-stage acral and cutaneous melanomas

Feature	DN(50 cases), n(%)	Melanoma(59cases), n(%)	*p*^a^ value
Gender ratio (male:female)	20:30 (0.67:1)	25:34 (0.74:1)	.802
Age, range (mean)	3–70 (36.60)	9–76 (47.73)	< .001*
Site			
Acral	31 (62.0)	42 (71.2)	.169
Trunk	9 (18.0)	6 (10.3)	
Limb	9 (18.0)	6 (10.2)	
Others	1 (2.0)	5 (8.5)	
mitotic figures≥1/mm^2^	1 (2.0)	23 (39.0)	< .001*
Diameter, range (mean, mm)	1–20 (7.30)	2–40 (11.34)	.002*
Stratum corneum pigmentation	17 (34.0)	38 (64.4)	.002*
Moderate to severe lymphocyte infiltration	6 (12.0)	28 (47.5)	< .001*
Moderate to severe cell atypia	6 (12.0)	52 (88.1)	< .001*
Melanocyte growth pattern in the epidermis			
Concessive distribution	20 (40.0)	48 (81.4)	< .001*
Pagetoid spread	2 (4.0)	20 (33.9)	< .001*
Nested distribution	33 (66.0)	44 (74.6)	.327
Scattered distribution	37 (74.0)	26 (44.1)	.002*

Abbreviations: *DN* dysplastic nevus, *n* number

^a^*p* values from Pearson’s chi-square test, independent *t*-test. *p < .05, considered statistically significant

Several features were considerably more frequently observed in the melanoma group, including more than one mitotic figures per square millimeter (39.0% vs. 2.0%, *p* < .001), stratum corneum pigmentation (64.4% vs. 34.0%, *p* = .002), moderate to severe lymphocyte infiltration (47.5% vs. 12.0%, *p* < .001), moderate to severe cell atypia (88.1% vs. 12.0%, p < .001), and a successive (81.4% vs. 40.0%, p < .001) or pagetoid (33.9% vs. 4.0%, p < .001) melanocyte growth pattern in the epidermis. Maturation of melanocytes was analyzed between specimens of compound nevi and invasive melanomas. The results revealed that all the 22 melanomas were immature, and among the 28 compound nevi specimens, only 3 were immature (100% vs. 10.71%, *p* < .001).

### FISH detection

**Fig. 1:**
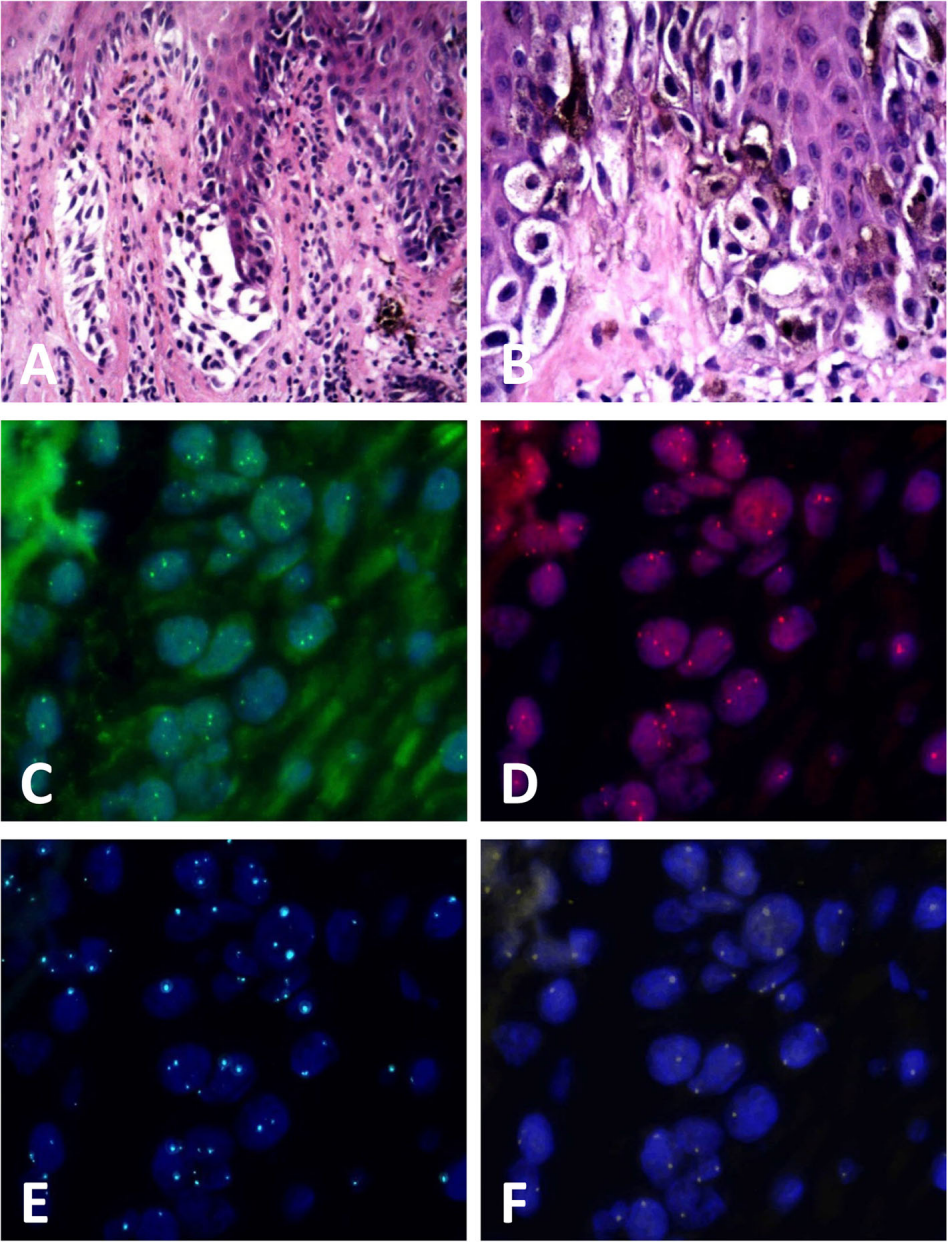
Case of melanoma in situ positive by fluorescence in-situ hybridization (FISH) detection. **a** Melanocytes proliferated successively or in nests in the basal layer of the epidermis (hematoxylin and eosin, H&E staining), (**b**) high-power magnification showed moderate to severe atypia of the melanocytes(H&E staining), (**c-f**) FISH images showed the (**c**) gain of *CCND1* (green signals), (**d**) gain of *RREB1* (red signals), and (**f**) loss of *MYB* (gold signals) relative to *CEP6* (aqua signals). While the gain of *RREB1*(**d**) relative to *CEP6* (**e**) was not observed

**Fig. 2:**
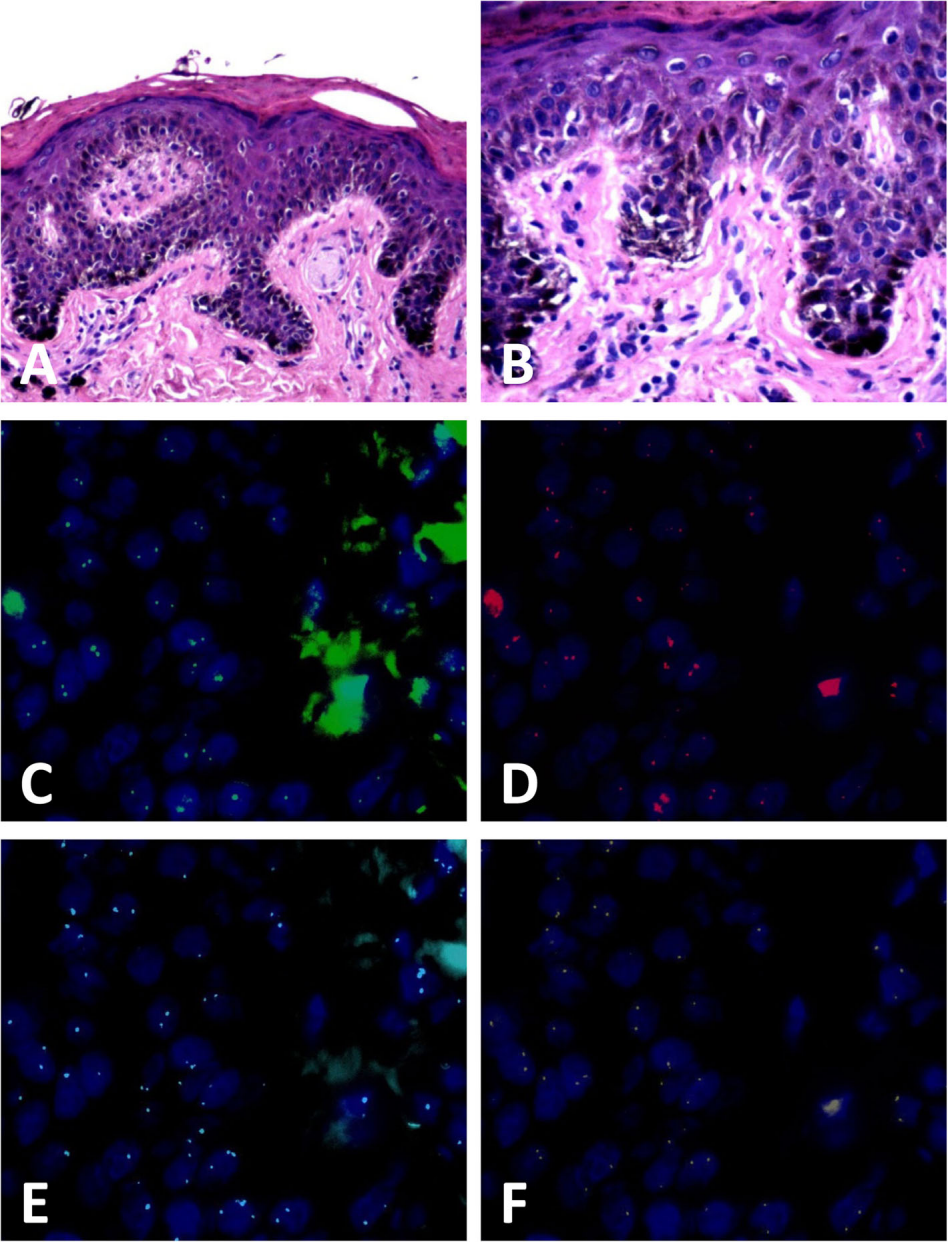
Case of dysplastic junctional nevus negative by fluorescenc e in-situ hybridization (FISH) detection. **a** Melanocytes proliferated successively or scattered in the basal layer of the epidermis (hematoxylin and eosin, H&E staining), (**b**)high-power magnification showed mild atypia of the melanocytes(H&E staining), (**c-f**) FISH images showed normal copies of *CCND1* (**c**, green signals), *RREB1* (**d**, red signals), *CEP6* (**e**, aqua signals), and *MYB* (**f**, gold signals)

**Fig. 3:**
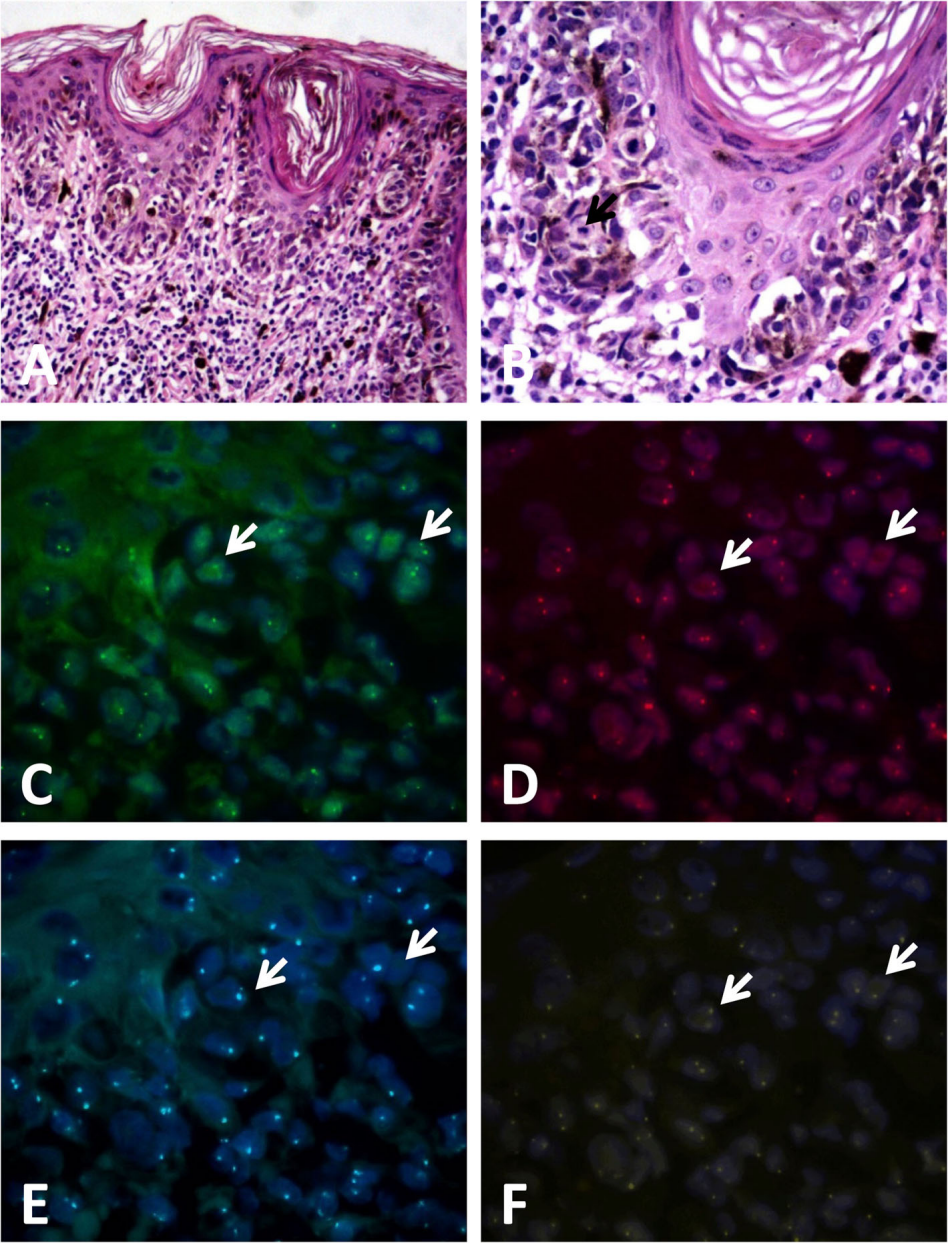
Case of melanoma in situ negative by fluorescence in-situ hybridization (FISH) detection. **a** Melanocytes proliferated successively or in nests in the basal layer of the epidermis (hematoxylin and eosin, H&E staining),(**b**) high-power magnification showed moderate atypia of the melanocytes with mitotic figures, which were easy to identify (black arrow)(H&E staining), (**c-f**) FISH images showed normal copies of*CCND1* (**c**, green signals), *RREB1* (**d**, red signals), *CEP6* (**e**, aqua signals), and *MYB* (**f**, gold signals) in tumor cells (white arrows)

**Fig. 4:**
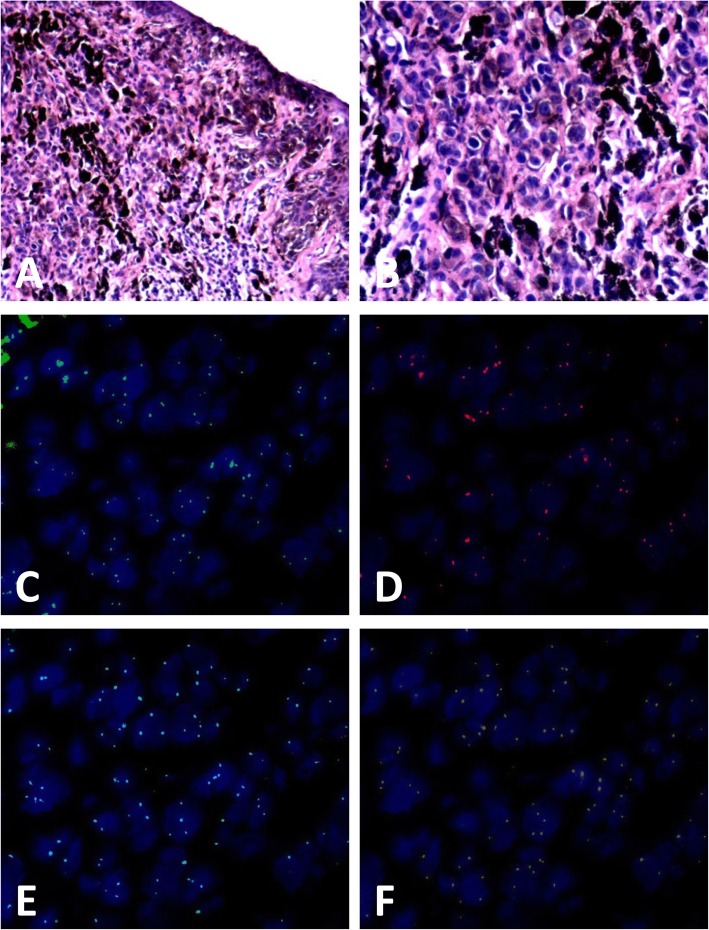
Case of compound nevus positive by fluorescence in-situ hybridization (FISH) detection. **a** Melanocytes scattered in the basal layer of the epidermis and proliferated without maturation in the dermis (hematoxylin and eosin, H&E staining), (**b**) high-power magnification showed mild atypia of the melanocytes and no mitotic Fig. (H&E staining),(**c-f**) FISH images showed the (**c**) gain of *CCND1* (green signals), (**d**) gain of *RREB1* (red signals),while the gain of *RREB1*(**d**, red signals) relative to *CEP6* (**e**, aqua signals) or loss of *MYB* (**f**, gold signals) relative to *CEP6* (**e**, aqua signals) was not observed

The results of FISH was positive in 3 (3/50, 6.0%) DN and 56 (56/59, 94.9%) melanoma cases (showed in Figs. 1, 2, 3 and 4) according to Gerami’s criteria. In distinguishing early-stage melanoma from DN, the sensitivity of FISH was 94.9%, specificity was 94.0%, positive predictive value was 94.9%, and negative predictive value was 94.0%. Two cases of melanomas primarily misdiagnosed as nevus turned out to be positive and two cases of DNs primarily misdiagnosed as melanoma were negative in FISH detection.

Among the 56 melanoma cases positive by FISH detection, only one case fulfilled all four criteria of Gerami, and nearly half of them fulfilled three criteria, specifically, 18 cases met the criteria “gain of *CCND1*”, “gain of *RREB1*”,and “loss of *MYB* relative to *CEP6*”, and 8 cases met “gain of *RREB1*”, “gain of *CCND1*”, and “gain of *RREB1* relative to *CEP6*”. Most of the remaining cases satisfied two of the criteria, with 15 of them satisfying “gain of *CCND1*” and “gain of *RREB1*”, 4 of them meeting “gain of *CCND1*” and “loss of *MYB* relative to *CEP6*”, and 1meeting both “gain of *RREB1*” and “loss of *MYB* relative to *CEP6*”. While 9 cases fulfilled only one criterion, that is, 8 cases were positive for “gain of *CCND1*”, and one positive for “gain of *RREB1*”. Of the 3DN positive cases by FISH assay, all fulfilled both “gain of *RREB1*” and “gain of *CCND1*”, and one of them fulfilled the criterion of “loss of *MYB* relative to *CEP6*” as well.

We also analyzed the number and percentage of cases meeting each criterion of Gerami and Gaiser (Table 2). The results showed that the criterion associated with the gain of *CCND1*was the most frequent to fulfill in the two criterion systems, with the positive rate of 91.5 and 88.1%, respectively, in the melanoma group and both 6.0% in the DN group, followed by Gerami criterion “gain of *RREB1*” with the positive rate of 74.6% in the melanoma group.

**Table 2:** Number and percentage of cases meeting each diagnostic criterion of Gerami and Gaiser for dysplastic nevi and early-stage acral and cutaneous melanomas

	DN, n(%)	melanoma, n(%)
Total, n	50	59
Gerami’s criteria		
RREB1 > CEP6 (> 55%)	0	9 (15.3)
RREB1 > 2 (> 29%)	3 (6.0)	44 (74.6)
MYB<CEP6 (> 40%)	1 (2.0)	24 (40.7)
CCND1 > 2 (> 38%)	3 (6.0)	54 (91.5)
FISH positive	3 (6.0)	56 (94.9)
Gaiser’s criteria		
RREB1 aberrant (> 63%)	3 (6.0)	28 (47.5)
MYB<CEP6 (> 31%)	1 (2.0)	30 (50.8)
MYB ≥ 2.5	3 (6.0)	28 (47.5)
CCND1 ≥ 2.5	3 (6.0)	52 (88.1)
FISH positive	5 (10.0)	55 (93.2)

Abbreviations: *DN* dysplastic nevus, *n* number

The sensitivity and specificity of each criterion of Gerami and Gaiser were studied (Table 3). According to Gerami’s criteria, the most sensitive criterion was “gain of *CCND1*” (91.5%), followed by “gain of *RREB1*”(74.6%), and the most specific criterion was “gain of *RREB1* relative to *CEP6*” (100%). If FISH was interpreted with Gaiser’s criteria, the overall sensitivity was 93.2% and specificity was 90.0%, and the most sensitive criterion was the gain of *CCND1*, which was 88.1%; the sensitivity of the other three criteria was very close.

**Table 3:** Sensitivity and specificity of each criterion of Gerami and Gaiser in differentiating dysplastic nevi and early-stage acral and cutaneous melanomas

	Sensitivity	Specificity
Gerami’s criteria		
RREB1 > CEP6 (> 55%)	15.2%	100%
RREB1 > 2 (> 29%)	74.6%	94.0%
MYB<CEP6 (> 40%)	40.7%	98.0%
CCND1 > 2 (> 38%)	91.5%	94.0%
Total^a^	94.9%	94.0%
Gaiser’s criteria		
RREB1 aberrant (> 63%)	47.5%	94.0%
MYB<CEP6 (> 31%)	50.8%	98.0%
MYB ≥ 2.5	47.5%	94.0%
CCND1 ≥ 2.5	88.1%	94.0%
Total^a^	93.2%	90.0%

^a^ total sensitivity and specificity of the four criteria, fulfilling any of the four criteria is considered positive

We compared the differences in *CCND1* amplification between the acral and cutaneous groups of melanoma (Table 4) and showed that *CCND1*was more amplified in the acral group. Specifically, the mean *CCND1* copy number per cell and percentage of cells with *CCND1* amplification were considerably higher in the acral melanoma group, with the average of 3.74–3.03 (*p* = .073) and 76.74–58.67% (*p* = .007), respectively. Cases meeting the criteria associated with *CCND1*amplification were also more in the acral group, although the differences were not so obvious.

**Table 4:** Comparison of the number and percentage of cases meeting each criterion of Gerami and Gaiser, and CCND1 amplification between the acral and non-acral groups of melanoma

	Acral group, n(%)	Non-acral group, n(%)	*p*^a^ value
Total	42	17	
Gerami’s criteria			
RREB1 > CEP6 (> 55%)	6 (14.3)	3 (17.6)	1.000
RREB1 > 2 (> 29%)	33 (78.6)	11 (64.7)	.437
MYB<CEP6 (> 40%)	19 (45.2)	5 (29.4)	.262
CCND1 > 2 (> 38%)	40 (95.2)	14 (82.4)	.274
FISH positive	41 (97.6)	15 (88.2)	.197
Gaiser’s criteria			
RREB1 aberrant (> 63%)	22 (52.4)	6 (35.3)	.234
MYB<CEP6 (> 31%)	21 (50.0)	9 (52.9)	.838
MYB ≥ 2.5	21 (50.0)	7 (41.2)	.539
CCND1 ≥ 2.5	39 (92.9)	13 (76.5)	.187
FISH positive	40 (95.2)	15 (88.2)	.691
CCND1 copy numbers per cell,mean	3.74	3.03	.073
Percentage of cells with CCND1 > 2,mean	76.74%	58.67%	.007*

Abbreviations: *n* number

^a^*p* values from Pearson’s chi-square test, independent *t*-test. *p < .05, considered statistically significant

## Discussion

The results of the present study showed that the four-color FISH detection was highly sensitive and specific in differentiating early-stage melanoma from DN in Chinese population, and the gain of *CCND1*presented the highest sensitivity. To the best of our knowledge, this is the first study to analyze the usage of FISH focusing on early-stage melanomas in Chinese patients, and the majority of which were acral melanomas in situ.

The incidence of melanoma in East Asia and Southeast Asia is significantly lower than that in North America and Europe. According to the GLOBOCAN 2012 statistics, the incidence of age-standardized melanomas is 0.4–0.5/100000 persons in East and Southeast Asia, while 8.6–13.8/100000 persons in Europe and North America [14]. However, due to the huge population, the burden of melanoma should not be ignored in Asia. Moreover, in Asia, especially in China, melanoma is often neglected and diagnosed late, and therefore, patients with melanoma in these areas are usually in an advanced stage with poor prognosis [15, 16]. Therefore, an early diagnosis of melanoma is very important.

Histologically, early-stage melanoma and DN can overlap, especially in the acral area. For example, melanocytes in acral DN are usually solitary and show mild to moderate cell atypia and lymphocyte or histocyte infiltration [17]. On the contrary, early-stage acral melanoma mostly manifests as solitary atypical melanocytes in the dermo–epidermal junction area [18]. In this study, we found that moderate to severe cell atypia, successive or pagetoid melanocyte growth pattern in the epidermis, more than one mitotic figures per square millimeter, moderate to severe lymphocyte infiltration, and stratum corneum pigmentation were significantly more often observed in early-stage acral and cutaneous melanomas than in nevi of such areas. However, distinguishing DNs from early melanomas in areas of acral is still difficult. Most of the above features in favor of differential diagnosis were observed in only less than half of the melanoma group patients in our study. Furthermore, none of the features was specific enough to diagnose a melanoma.

Four-color FISH probe is a useful tool to assist the diagnosis of melanoma. The results of various studies are not consistent, with a sensitivity of 70.5–100% and specificity of 90–100% [3–11]. The specificity in our study was similar to that reported previously, but the sensitivity was considerably higher [8–11]. The differences in sensitivity might be associated with the site and ethnic differences, and tumor stage. Studies have shown significant differences in genotypes between acral and cutaneous melanomas, also among different ethnic populations [19–22].

We found that the *CCND1*gene gain was more common in the acral group than in the cutaneous group. This result was in accordance with those of studies based on comparative genomic hybridization and immunohistochemistry [22, 23]. However, the most sensitive criterion in our study was the gain of *CCND1*, which was considerably higher than that in previous studies of acral melanomas, including a study based on the same ethnic population, in which the majority of cases was advanced melanomas and the most sensitive criterion was the gain of *RREB1* instead [10, 24]. Interestingly, in Su’s study [10], of the seven cases of acral melanoma in situ, the most sensitive criterion was also the gain of *CCND1*, specifically, 5 cases were positive for *CCND1*; of the two negative cases, one showed 33% of cells gaining *CCND1* gene, close to the positive threshold of 38%, while the other one was negative in the FISH assays with all the probes, including the *MYC* and *CDKN2A* probes. Therefore, we inferred that the *CCND1*gene gain is one of the most frequent genetic changes in early-stage acral melanomas in the Chinese population, and it was helpful in diagnosing acral melanoma in situ. Studies have indicated frequent amplification of genes involved in the CDK4 pathway including *CCND1* in acral melanoma [21, 25], indicating the potential for CDK4/6 inhibitors. However, the significance of *CCND1* gain in the occurrence or in situ state of acral melanoma needs further studies.

Considering that false positivity and negativity still exited in a subset of cases, FISH detection should be supplemented with histopathological evaluation and should not replace conventional microscopy in discriminating melanomas from DNs. Three nevus cases were positive for the FISH assay in our study. Further analysis showed that all of the four genes (*CCND1*, *RREB1*,*MYB* and *CEP6*) manifested three copies, suspicious of polyploidy, which is the most common false positivity in spitz nevi. None of the three cases was spitz nevus in morphology, though. However, as it is possible that genetic changes might precede morphological changes, we recommend that for those DN cases with positive FISH results, complete resection with enough negative margin and close follow-up might be the appropriate treatment. Three melanoma cases with negative FISH results were found in this study. Since we only tested four genes, it is comprehensible to understand that these cases might be associated with other gene alterations such as *MYB* loss and *CDKN2A* homozygous deletion. A combination with *MYB* or *CDKN2A* probes may increase the sensitivity [10].

A limitation of the study is that we lacked prognosis information and regarded the histopathological diagnosis as the golden standard. Considering the good prognosis of early-stage melanoma, long time follow-up for decades is needed to elucidate its biological behavior, and maybe then we can investigate and distinguish the two groups more thoroughly. Another limitation is that we have not confirmed the gene alterations of the three false-negative melanoma cases with a MYC or CDKN2A probes or a 5 or 7 color FISH probes, since the former probes are unavailable for us.

## Conclusions

To conclude, with high sensitivity and specificity, the four-color FISH technique was a valuable ancillary tool to distinguish early-stage acral and cutaneous melanomas from DNs in Chinese population. Furthermore, the *CCND1* gene gain was the most sensitive criterion of melanomas in this cohort. A combination of the four-color FISH results with histological analysis was necessary to explain the positive or negative significance of FISH. For DN cases with positive FISH results, complete resection with sufficient negative margin and close follow-up might be the appropriate treatment.

## Data Availability

Not applicable.

## Abbreviations

DN: Dysplastic nevus
FISH: Fluorescence in-situ hybridization
H&E: Hematoxylin and eosin
